# Roles of Fhit and p53 in Taiwanese surgically treated non-small-cell lung cancers

**DOI:** 10.1038/sj.bjc.6601041

**Published:** 2003-07-15

**Authors:** Y-L Chang, C-T Wu, J-Y Shih, Y-C Lee

**Affiliations:** 1 Department of Pathology, National Taiwan University Hospital and National Taiwan University College of Medicine, Taipei, 100, Taiwan; 2 Department of Internal Medicine, National Taiwan University Hospital and National Taiwan University College of Medicine, Taipei, 100 Taiwan; 3 Department of Surgery, National Taiwan University Hospital and National Taiwan University College of Medicine, Taipei, 100 Taiwan

**Keywords:** Fhit, p53, immunohistochemistry, lung cancers

## Abstract

Abnormalities of fragile histidine triad (FHIT) and TP53 have been found frequently in nonsmall cell lung cancers. In the current study, 263 primary nonsmall cell lung cancers were investigated for the expressions of Fhit and p53 by immunohistochemistry. Marked reduction of Fhit immunoreactivity (<10% positivity) in 156 (59%) tumours and overexpression of p53 protein (>10% positivity) in 89 (34%) tumours were found. Reduced Fhit expression was also noted in most squamous cell carcinomas (80 out of 99, 81%), and in a smaller fraction of adenocarcinomas (76 out of 164, 46%; *P*<0.001). p53 nuclear staining was demonstrated in 54 out of 99 (55%) squamous cell carcinomas and in 35 out of 164 (21%) adenocarcinomas (*P*<0.001). The loss of Fhit expression and p53 overexpression was significantly more common in tumours occurring in smokers (93 out of 113, 82% and 56 out of 113, 50%) than in those of nonsmokers (63 out of 150, 42%; *P*<0.001 and 33 out of 150, 22%; *P*<0.001). Notably, p53 overexpression was associated with distant metastasis of patients in the whole series (*P*=0.027) and in adenocarcinoma (*P*=0.001). It was also associated with a poorer survival of patients with adenocarcinoma (*P*=0.032).

Lung cancer is the most common cause of cancer death in Taiwan (Lee *et al*, 2002). It is generally believed that cancer is the end result of a multistep process involving the activation of dominant oncogenes and the inactivation of tumour suppressor genes. Recent advances in the molecular genetics of human cancers have revealed that multiple tumour suppressor genes are involved in lung carcinogenesis (Yokota and Sugimura, 1993). The cloning of the fragile histidine triad(FHIT) gene at 3p14.2 in 1996 (Ohta *et al*, 1996) and the subsequent reports demonstrated frequent allelic deletion, aberrant FHIT transcripts in primary lung cancers (Fong *et al*, 1997), cell lines of small cell and nonsmall cell type (Yanagisawa *et al*, 1996). The evidence that FHIT suppresses tumorigenicity in cancer cells (Siprashvili *et al*, 1997) supports the contention that FHIT is a tumour suppressor gene. This, together with the recent observation (Sozzi *et al*, 1997a,Sozzi *et al*, 1997b) that there is more FHIT allelic loss in carcinomas from smokers than from nonsmokers, strengthens the case for its involvement in the multistage development of lung cancer.

TP53, on the other hand, is a well-established inducer of apoptosis and a major checkpoint protein involved in DNA, spindle, and centrosome surveillance. Alteration of TP53 genes has been among the most commonly observed tumour genetic changes that have been closely related to the initiation and progression of a diverse group of human cancers, including lung cancer (Levine *et al*, 1991). The prevalent type of mutation is also related to DNA adducts of benzo(*a*)pyrene from cigarette smoking (Denissenko *et al*, 1996).

There are several reports on the correlation between abnormalities of the FHIT and TP53 gene and clinicopathological features in lung cancers. Loss of heterozygosity (LOH) at the FHIT gene locus in adenocarcinoma was less frequent than that in squamous cell carcinoma (Burke *et al*, 1998). The correlation between LOH at the FHIT gene locus and the patients' survival is controversial (Fong *et al*, 1997; Sozzi *et al*, 1997a,1997b; Burke *et al*, 1998; Tomizawa *et al*, 1998). LOH at the FHIT gene locus in smokers occurred more frequently than that in nonsmokers (Sozzi *et al*, 1997a,1997b; Zienolddiny *et al*, 2001). It was also reported that there was no correlation between Fhit protein expression with the histotype, tumour stage, survival (Sozzi *et al*, 1997a,1997b; Geradts *et al*, 2000) and abnormalities of immunohistochemical expression of p53, Rb, and p16 (Geradts *et al*, 2000). However, further large studies of association of FHIT and TP53 alterations with clinical parameters are warranted.

Since immunohistochemical detection has the advantage of detecting protein loss regardless of the underlying mechanism, it represents an efficient method of identifying functional protein inactivation. In this study, the expression of Fhit and p53 protein by immunohistochemistry (IHC) in 263 surgically resected NSCLCs were investigated. These findings were correlated with the clinicopathological features, including smoking history, histologic types and differentiation, tumour size, stage, tumour emboli, tumour invasion, distant metastasis, and survival.

## MATERIALS AND METHODS

### Lung cancer patients and specimens

A total of 263 lung cancer specimens were obtained from patients who underwent surgical resection for NSCLC at the National Taiwan University Hospital for the period from January 1996 to December 2000. These patients were not treated with neoadjuvant chemotherapy and irradiation therapy. All the specimens were formalin fixed and sectioned for microscopic examination after applying haematoxylin–eosin stain. Histological diagnosis and pathological features were obtained, including tumour cell type, degree of differentiation, tumour emboli, direct invasion to surrounding structures, and regional lymph node metastasis. Pathological staging was performed according to the international staging system for lung cancer (Mountain, 1986), which is based on tumour size, location and involvement, and the presence of lymph node metastases.

This study included 143 male and 120 female patients, and the mean age was 64 years (ranging from 30 to 83 years). The clinical data of these patients, including sex, age, smoking status, location of the tumour, and ensuing distant metastases after surgery were recorded, and correlated with the result of Fhit or p53 protein expression in each tumour. No information was available for distant metastasis in nine patients, and for tumour size and stage in one patient. The 15-stage IIIB cases included 12 multifocal carcinomas within one pulmonary lobe, two cancers with pulmonary artery and aortic invasions, and one with mediastinal involvement. All the nine stage IV cases were multifocal carcinomas involving two pulmonary lobes.

### Immunohistochemistry

For immunohistochemical demonstration of the Fhit or p53 protein expression in the tumour tissue, 4-*μ*m-thick sections from each formalin-fixed, paraffin-embedded tissue block were dewaxed with xylene and rehydration through a graded series of ethanol.

The sections for IHC of the Fhit protein expression were autoclaved in 0.01 M phosphate citrate buffer (pH 6.0) at 121°C for 3 min and were treated with 3% H_2_O_2_–methanol solution to reduce endogenous peroxidase activity. These were then incubated with normal goat serum to reduce nonspecific antibody binding and were subsequently subjected to the primary antibody reaction. The antibody for Fhit protein (IBL, Gunma, Japan, 1 : 20) was left to react with the sections overnight at 4°C. Detection of the immunoreactive staining was carried out by the avidin–biotin–peroxidase complex method according to the manufacturer's instructions (Dako Corporation, Carpinteria, CA, USA). To check for nonspecific staining by the avidin–biotin–peroxidase complex detection system, the primary antibody was replaced with BSA. The sections were then subjected to a colour reaction with 0.05% 3,3-diaminobenzidine in 0.05 M trishydrochloride (pH 7.6) containing 0.01% H_2_O_2_ and were lightly counterstained with haematoxylin.

For IHC of the p53 protein expression in the tumour tissue, the paraffin-embedded tissue block was treated with 0.3% H_2_O_2_ in methanol to block endogenous peroxidase, and heated in a microwave oven for 20 min for antigen retrieval. The tissue sections were then incubated with normal nonimmune goat serum. After blotting the excessive goat serum, the slides were incubated with a specific mouse anti-p53 protein antibody ‘p53 (Ab-6), pantropic’ (diluted 1 : 50) (Oncogene Science, Cambridge, MA, USA) for 1 h at room temperature. After washing with a phosphate-buffered solution (PBS) three times, the sections were incubated with biotinylated goat anti-mouse antibody for 20 min at room temperature. The sections were again washed three times with PBS, and were then incubated with peroxidase-conjugated steptavidin for 15 min at room temperature. After a third triple washing with PBS, the sections were then stained with 0.05% 3′,3-diaminobenzidine tetrachloride freshly prepared in 0.05 M Tris-HCl (pH=7.6) containing 0.01% H_2_O_2_. Finally, the sections were counterstained with haematoxylin and then mounted.

Immunostaining was classified into the following three groups according to both intensity and extent: (1) negative, no staining was present, or positive staining was detected in <10% of the cells; (2) positive staining in a range of 10–50% of cells stained; and (3) positive immunostaining was present in >50% of the cells. Two independent pathologists (Y-LC and C-TW) were involved in the assessment of expression.

### Statistical analysis

The correlation between various clinical or pathological parameters with the expression of Fhit or p53 protein was analysed using Pearson's *χ*^2^, Fisher's exact, and log-rank tests. All the statistical tests were two sided.

## RESULTS

### Fhit protein expression

**Figure 1:**
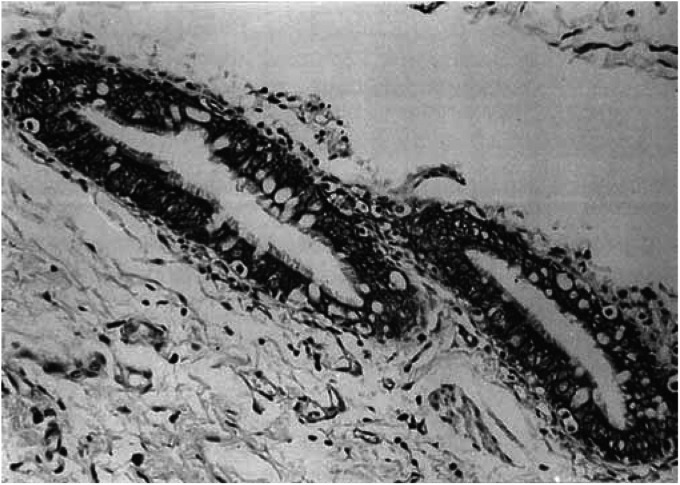
Normal bronchial epithelium used as a positive control of Fhit protein (original magnification × 66; ABC method).

In the current study, Fhit cytoplasmic reactivity was detected in 107 out of the 263 lung cancers (41%), usually of moderate-to-strong intensity. The remaining 156 lung cancers (59%) were characterised by complete absence of cytoplasmic staining or marked reduction (<10%) of immunoreactivity. Admixed non-neoplastic elements (normal bronchial epithelial cells, bronchial glands, and type II alveolar cells) served as Fhit-positive internal controls (Figure 1

). The relationship between clinical parameters or pathological characteristics, and the frequency of Fhit reactivity is shown in Table 1

**Table 1:** Frequency of Fhit expression with relation to clinical parameters and pathologic characteristics

**Parameters**	**No. of patients**	**Fhit (−) (%)**	**Fhit (+) (%)**	***P*-value**
Patient number	263	156 (59)	107 (41)	

Sex				
Male	143	100 (70)	43 (30)	
Female	120	56 (47)	64 (53)	<0.001

Smoking history				
Positive	113	93 (82)	20 (18)	
	(male 103; female 10)			
Negative	150	63 (42)	87 (58)	<0.001
	(male 40; female 110)			

Histologic type				
Squamous cell carcinoma	99	80 (81)	19 (19)	
Adenocarcinoma	164	76 (46)	88 (54)	<0.001

Squamous cell carcinoma				
Poorly differentiated	10	8 (80)	2 (20)	
Others	89	72 (81)	17 (19)	1.000^a^

Adenocarcinoma				
Poorly differentiated	9	7 (78)	2 (22)	
Others	155	69 (45)	86 (55)	0.083^a^

Tumour size				
3 cm and less	120	64 (53)	56 (47)	
>3 cm	142	91 (64)	51 (36)	0.078

Tumour stage				
Stage I	124	71 (57)	53 (43)	
Stages II and IIIA	114	67 (59)	47 (41)	
Stages IIIB and IV	24	17 (71)	7 (29)	0.449

Tumour emboli				
Positive	58	36 (62)	22 (38)	
Negative	205	120 (59)	85 (41)	0.629

Tumour local invasion				
Positive	62	35 (56)	27 (44)	
Negative	201	121 (60)	80 (40)	0.600

Distant metastasis				
Positive	99	60 (61)	39 (39)	
Negative	155	88 (57)	67 (43)	0.546

p53 expression				
p53 (+)	89	61 (69)	28 (31)	
p53 (−)	174	95 (55)	79 (45)	0.030

Distant metastasis in adenocarcinomas				
Positive	65	33 (51)	32 (49)	
Negative	97	42 (43)	55 (57)	0.350

a Fisher's exact test.

Others: Pearson's *χ*^2^test.

.

**Figure 2:**
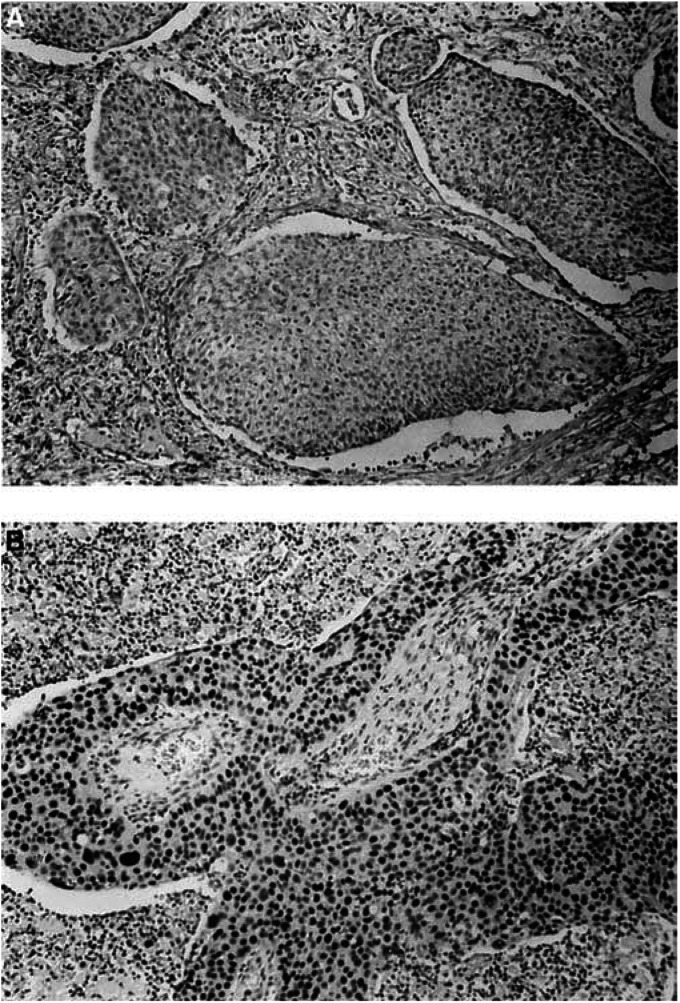
Squamous cell carcinoma of a smoker. (**A**) Loss of Fhit expression (original magnification × 33; ABC method). (**B**) Overexpression of nuclear staining for p53 (original magnification × 33; ABC method).

Fhit expression was markedly reduced in most squamous cell carcinomas (80 out of 99, 81%) (Figure 2A

). A similar loss of expression was detected in a smaller subset of adenocarcinoma (76 out of 164, 46%). The difference in the frequency of loss of Fhit expression between squamous cell carcinomas and adenocarcinomas was statistically significant (*P*<0.001). A loss of Fhit protein expression was observed more frequently in patients with a smoking history (93 out of 113, 82%) than in patients without a smoking history (63 out of 150, 42%), and such a difference was also statistically significant (*P*<0.001).

**Figure 3:**
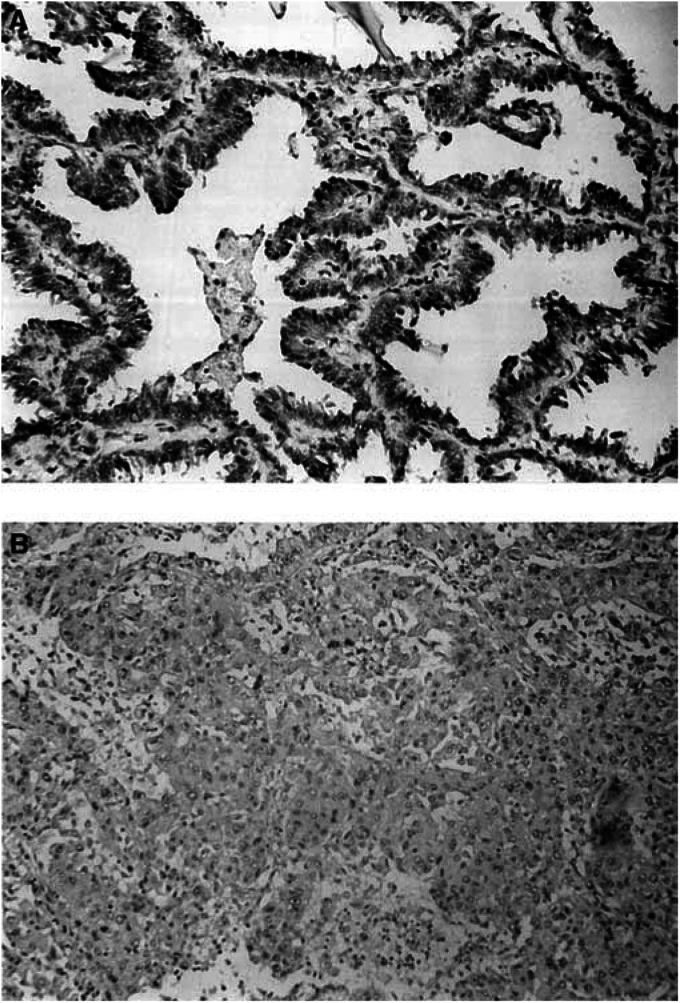
Fhit immunoreactivity in adenocarcinoma. (**A**) Strong Fhit cytoplasmic immunoreactivity in a well-differentiated adenocarcinoma (original magnification × 66; ABC method). (**B**) Weak or negative Fhit immunoreactivity in the less differentiated adenocarcinoma (original magnification × 33; ABC method).

The correlation between Fhit protein expression and differentiation of adenocarcinoma (Figure 3A

) and squamous cell carcinoma subtypes were then analysed. In adenocarcinoma, the loss of expression was observed preferentially in poorly differentiated subtypes (Figure 3B); however, the correlation did not reach statistical significance (*P*=0.083). In squamous cell carcinoma, the correlation between Fhit protein expression and differentiation was unclear (*P*=1.000).

Furthermore, Fhit expression was also prominently lost in 100 out of 143 (70%) male patients and 56 out of 120 (47%) female patients. This difference was statistically significant (*P*<0.001). However, in this cohort of 263 Taiwanese NSCLCs, there was no statistically significant correlation between the loss of Fhit expression and tumour size, stage of disease, distant metastasis, tumour emboli, direct invasion to surrounding structures, and survival (Table 1).

### p53 protein expression

Overexpression of p53 protein (>10% nuclear staining) was observed in 89 out of 263 cases (34%). The relationship between clinical parameters or pathological characteristics, and the frequency of p53 reactivity is shown in Table 2

**Table 2:** Frequency of p53 expression with relation to clinical parameters and pathologic characteristics

**Parameters**	**No. of patients**	**P53 (+) (%)**	**P53 (−) (%)**	***P*-value**
Patient number	263	89 (34)	174 (66)	

Sex				
Male	143	63 (44)	80 (56)	
Female	120	26 (22)	94 (78)	<0.001

Smoking history				
Positive	113	56 (50)	57 (50)	
	(Male 103; Female 10)			
Negative	150	33 (22)	117 (78)	<0.001
	(Male 40; Female 110)			

Histologic type				
Squamous cell carcinoma	99	54 (55)	45 (45)	
Adenocarcinoma	164	35 (21)	129 (79)	<0.001

Squamous cell carcinoma				
Poorly differentiated	10	6 (60)	4 (40)	
Others	89	48 (54)	41 (46)	0.752^a^

Adenocarcinoma				
Poorly differentiated	9	1 (11)	8 (89)	
Others	155	34 (22)	121 (78)	0.686^a^

Tumour size				
3 cm and less	120	41 (34)	79 (66)	
>3 cm	142	48 (34)	94 (66)	0.951

Tumour stage				
Stage I	124	41 (33)	83 (67)	
Stages II and IIIA	114	42 (37)	72 (63)	
Stages IIIB and IV	24	6 (25)	18 (75)	0.515

Tumour emboli				
Positive	58	21 (36)	37 (64)	
Negative	205	68 (33)	137 (67)	0.666

Tumour local invasion				
Positive	62	22 (35)	40 (65)	
Negative	201	67 (33)	134 (67)	0.754

Distant metastasis				
Positive	99	40 (40)	59 (60)	
Negative	155	42 (27)	113 (73)	0.027

Distant metastasis in adenocarcinomas				
Positive	65	22 (34)	43 (66)	
Negative	97	12 (12)	85 (88)	0.001

a Fisher's exact test.

Others: Pearson *χ*^2^test.

**Figure 4:**
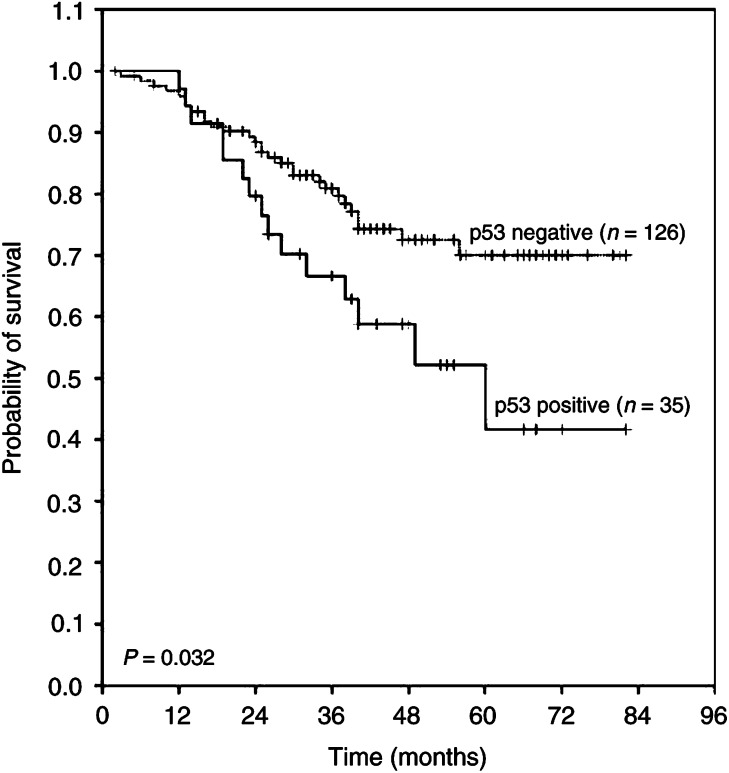
Kaplan–Meier survival curves of p53-positive and p53-negative adenocarcinoma patients after surgery (*P*=0.032). (All patients alive on their last follow-up are indicated by tick marks.)

. p53 had significantly higher positive results in patients with squamous cell carcinoma subtype (54 out of 99, 55%) (Figure 2B). On the other hand, positivity was demonstrated only in 35 out of 164 (21%) of adenocarcinomas (*P*<0.001). Using Pearson's *χ*^2^ test, the difference of p53-positive staining between smokers (56 out of 113, 50%) and nonsmokers (33 out of 150, 22%) is significant statistically (*P*<0.001). p53 overexpression was also noted in 63 out of 143 (44%) male and 26 out of 120 (22%) female patients (*P*<0.001). In addition, p53 had significantly higher positive results in patients with distant metastasis (*P*=0.027) in the overall NSCLCs and in the adenocarcinoma subset (*P*=0.001) (Table 2), as well as a poorer survival of patients with adenocarcinoma (log-rank test *P*=0.032) (Figure 4

).

### Correlation of Fhit expression with p53 protein expression abnormalities

The correlation between Fhit and p53 protein expression has not been previously studied in detail. These results indicate that FHIT and TP53 gene alterations by Pearson's *χ*^2^ test are concordant and preferentially occur in squamous cell carcinomas, among smokers, and in male patients (Table 1 and 2).

## DISCUSSION

It is well documented that specific genetic alteration and aberrant gene expression in tumour cells may influence its biological behaviour (Levine *et al*, 1991). The genomic status of FHIT and TP53 genes has been investigated in a series of lung cancers. Deletions at the chromosomal region 3p14.2 and TP53 abnormalities were present in 20% of the cases and the alterations were constantly associated (Marchetti *et al*, 1998). Recently, it was reported that alterations in the FHIT locus detected by DNA and/or reverse transcription–PCR analysis correlated with a loss of Fhit protein expression in lung, cervical, and breast carcinomas (Greenspan *et al*, 1997; Sozzi *et al*, 1997a,1997b; Campiglio *et al*, 1999). The results indicated that FHIT gene alterations could easily be detected by immunohistochemical analysis of tumour specimens.

Altered Fhit expression is the most frequent genetic change in lung tumours. No major impact of Fhit expression on prognosis and other clinical parameters were observed. This finding indicates that the inactivation of the FHIT gene is involved in the early phases of lung carcinogenesis and hence, is more likely related to the initiation of the neoplastic process rather than to the progression, invasiveness, or distant metastasis.

TP53 is a tumour suppressor gene, involved in cell cycle control and the preservation of genomic integrity (Levine *et al*, 1991; Zambetti *et al*, 1993). Alteration of the TP53 gene may be involved in the initiation, development, progression, and invasion of lung cancer (Caamano *et al*, 1991; Sozzi *et al*, 1992; Marchetti *et al* 1993; Fontanini *et al*, 1994). Even though correlation of p53 protein expression with clinical and biological characteristics of lung cancer has been extensively studied, the results of these studies are quite diverse (Top *et al*, 1995; Passlick *et al*, 1995; Dalquen *et al*, 1996; Lee *et al* 1999; Garinis *et al*, 2001; Zienolddiny *et al*, 2001). The results of this study indicated that p53 expression was closely related to distant metastasis of the overall NSCLCs and the adenocarcinoma subtype; and p53 was especially useful as a prognostic factor in adenocarcinoma. This observation had never been reported before.

Fhit protein expression was markedly reduced in most squamous cell carcinomas, and such a reduction was significantly more frequent in the squamous cell carcinoma subtype than in the adenocarcinoma subtype. Furthermore, a marked reduction of Fhit protein expression was observed more frequently in patients with positive smoking histories than in patients with negative smoking histories. These data indicate that FHIT gene alterations preferentially occur in squamous cell carcinomas and in smokers. On the other hand, p53 overexpression is also significantly more common in tumours occurring in smokers than in those of nonsmokers. This constant association of Fhit and p53 abnormalities in tumours from smoking subjects is intriguing.

Loss of Fhit expression appears to be strongly associated with smoking status, suggesting that the FHIT gene may be the target of carcinogens in cigarette smoke. The study by Sozzi *et al* (1997a),1997b indicates that FRA3B is a preferential target of tobacco smoke damage at a molecular level. This suggests that loss of Fhit expression is due to damage at the fragile locus, resulting in FHIT loss. According to Burke *et al* (1998), the statistical associations among FHIT allelic deletion and TP53 missense mutations might be explained by the damage caused by tobacco smoke carcinogens. This is shown by the fact that TP53 mutations are more common in centrally located squamous cell carcinomas than in the peripheral adenocarcinomas (Greenblatt *et al*, 1994). A plausible explanation is the presence of an increased dosage of chemical carcinogens in the central airways compared with those present in peripheral lung tissues.

*In vitro* evidence for chemical carcinogenesis models linking carcinogen–DNA adducts to hotspots of TP53 missense mutations in human lung cancer has recently been provided (Denissenko *et al*, 1996), and analysis of human cancer cell deletion end points shows that the FRA3B fragile site is a common target of homologous recombination producing FHIT internal deletions (Inoue *et al*, 1997). Taken together, these results provide a direct link between specific genetic alterations and exposure to tobacco carcinogens. As in the reports mentioned before (Marchetti *et al*, 1998; Geradts *et al*, 2000), Fhit expression was not correlated to the abnormality of p53 in smoking patients. A recent study (Zienolddiny *et al*, 2001) indicated that LOH at 3p, 5q, 9p, 11p, and 17p regions were interdependent and highly associated with mutated TP53 gene in smokers. The significant association of Fhit and p53 protein expressions with smokers in the present study is perhaps related to the size of this series.

It is also noted that a loss of Fhit protein expression and p53 overexpression was significantly associated with male patients. The meaning of this association is not clear and has never been reported before. The possible explanation for such a phenomenon is that the smokers in our series are nearly all male patients and only 10 female patients were smokers. Thus, additional evaluation of Fhit and p53 protein expressions performed in a series with more female smoking patients would clarify this relationship in the future.

Interestingly, a reduction of Fhit expression was more commonly detected in the less differentiated areas of the adenocarcinoma than in the more differentiated cells . Although this difference did not have statistical significance, this observation is still suggestive of the possibility that Fhit protein expression is related to the degree of differentiation or the stage of progression in the adenocarcinoma subtype (Tomizawa *et al*, 1998).

In conclusion, this study is the first to demonstrate the concurrent loss of Fhit expression and p53 overexpression in squamous cell carcinoma and smokers by IHC. These alterations in expression also suggest a role in the initiation of smoking-related lung tumourigenesis. Likewise, there is an association between the pattern of Fhit expression and the level of cell differentiation in adenocarcinoma. Interestingly enough, the present results also have clinical implications due to the observation of a statistically significant trend towards distant metastasis and poorer survival in adenocarcinoma patients with p53 overexpression. Thus, it is strongly indicated that p53 overexpression could be a useful prognostic marker for surgically resected adenocarcinoma.
